# P5CDH affects the pathways contributing to Pro synthesis after ProDH activation by biotic and abiotic stress conditions

**DOI:** 10.3389/fpls.2015.00572

**Published:** 2015-07-28

**Authors:** Yanina S. Rizzi, Mariela I. Monteoliva, Georgina Fabro, Carola L. Grosso, Laura E. Laróvere, María E. Alvarez

**Affiliations:** ^1^Centro de Investigaciones en Química Biológica de Córdoba CIQUIBIC, UNC-CONICET, Departamento de Química Biológica, Facultad de Ciencias Químicas, Universidad Nacional de CórdobaCórdoba, Argentina; ^2^Centro de Estudio de las Metabolopatías Congénitas, Hospital de Niños de la Santísima Trinidad, Cátedra de Clínica Pediátrica, Facultad de Ciencias Médicas, Universidad Nacional de CórdobaCórdoba, Argentina

**Keywords:** Δ1 pyrroline-5-carboxylate dehydrogenase (P5CDH), proline dehydrogenase (ProDH), exogenous proline treatment, hypersensitive response, Pro, Orn, Glu, cell death

## Abstract

Plants facing adverse conditions usually alter proline (Pro) metabolism, generating changes that help restore the cellular homeostasis. These organisms synthesize Pro from glutamate (Glu) or ornithine (Orn) by two-step reactions that share Δ^1^ pyrroline-5-carboxylate (P5C) as intermediate. In the catabolic process, Pro is converted back to Glu using a different pathway that involves Pro dehydrogenase (ProDH), P5C dehydrogenase (P5CDH), and P5C as intermediate. Little is known about the coordination of the catabolic and biosynthetic routes under stress. To address this issue, we analyzed how P5CDH affects the activation of Pro synthesis, in Arabidopsis tissues that increase ProDH activity by transient exposure to exogenous Pro, or infection with *Pseudomonas syringae* pv. *tomato*. Wild-type (Col-0) and *p5cdh* mutant plants subjected to these treatments were used to monitor the Pro, Glu, and Orn levels, as well as the expression of genes from Pro metabolism. Col-0 and *p5cdh* tissues consecutively activated *ProDH* and Pro biosynthetic genes under both conditions. However, they manifested a different coordination between these routes. When external Pro supply was interrupted, wild-type leaves degraded Pro to basal levels at which point Pro synthesis, mainly *via* Glu, became activated. Under the same condition, *p5cdh* leaves sustained *ProDH* induction without reducing the Pro content but rather increasing it, apparently by stimulating the Orn pathway. In response to pathogen infection, both genotypes showed similar trends. While Col-0 plants seemed to induce both Pro biosynthetic routes, *p5cdh* mutant plants may primarily activate the Orn route. Our study contributes to the functional characterization of P5CDH in biotic and abiotic stress conditions, by revealing its capacity to modulate the fate of P5C, and prevalence of Orn or Glu as Pro precursors in tissues that initially consumed Pro.

## Introduction

Proline (Pro) metabolism is intimately associated with stress adaptation. Most plants accumulate Pro under drought, salinity, extreme temperatures, UV radiation, or pathogen infection. The levels reached by this compound depend on the plant species and the type of stress (Verslues and Sharma, 2010; Liang et al., 2013). Once stress is released Pro undergoes oxidative degradation, providing nitrogen, and energy for the recovery process. Stress-induced Pro accumulation is often accompanied by increased synthesis and reduced catabolism (Verslues and Sharma, 2010), but could also coexist with activation of both processes (Fabro et al., 2004; Kaplan et al., 2007). The Pro metabolic changes occurring under stress help alleviate cell damage in different ways. Due to its chemical nature, this amino acid can function as osmolyte, ROS scavenger and protein chaperone. Beyond this, its synthesis and degradation generates an extensive transport of intermediates and cofactors between different subcellular compartments, helping to adjust the cytosolic pH and cellular redox status (Liang et al., 2013; Ben Rejeb et al., 2014). Moreover, the Pro synthesized in shoots can be transported to roots for subsequent degradation, sustaining root growth under unfavorable conditions (Sharma et al., 2011). Interestingly, stress tolerance does not necessarily require Pro accumulation and even more, Pro degradation is needed for plant defense activation under some adverse conditions. This has been observed in Arabidopsis tissues developing the Hypersensitive Response (HR) triggered by the bacterial pathogen *Pseudomonas syringae* pv. *tomato* DC3000 *AvrRpm1 (Pst*-*AvrRpm1*). In this case, the limiting enzyme in Pro catabolism potentiates the oxidative burst and cell death associated to the HR program (Cecchini et al., 2011). Similarly, low doses of exogenous Pro activate the catabolic route, improving tolerance to salt, UV radiation, and temperature stress by alleviating cytoplasmic acidosis and redox alterations (Hayat et al., 2012).

**Figure 1:**
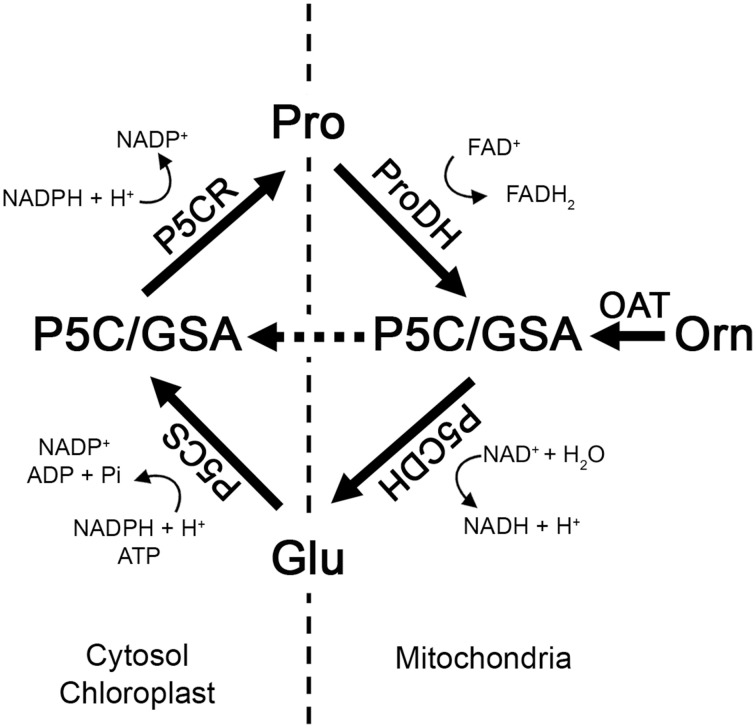
**Model of Pro metabolism in Arabidopsis**. P5CS and P5CR synthesize Pro from Glu at cytosol and chloroplasts. ProDH and P5CDH oxidize Pro into Glu at mitochondria. OAT transfers the delta amino group from Orn to 2-oxoglutarate producing GSA/P5C and Glu. P5C acts as an intermediate of all these pathways. GSA, glutamate-semialdehyde; P5C, Δ1 pyrroline-5-carboxylate; P5CS, P5C synthase; P5CR, P5C reductase; ProDH, proline dehydrogenase; P5CDH, P5C dehydrogenase; OAT, ornithine δ-amino transferase.

Most enzymes of Pro metabolism are well characterized at the biochemical level, but connections between anabolic and catabolic processes have not been thoroughly studied, particularly under circumstances where initial Pro degradation contributes to stimulate plant defenses. The Pro catabolic pathway takes place in mitochondria, where Pro is oxidized to glutamic acid (Glu) in two steps (Verslues and Sharma, 2010) (Figure 1). First, Pro dehydrogenase (ProDH) transforms Pro into Δ1 pyrroline-5-carboxylate (P5C), and then P5C dehydrogenase (P5CDH) converts the tautomeric form of P5C (glutamate-semialdehyde; GSA) into Glu. In turn, Glu can be used as precursor of Pro synthesis at cytosol and plastids. There, P5C synthase (P5CS) reduces Glu to GSA/P5C, and P5C reductase (P5CR) converts P5C into Pro (Funck et al., 2012). ProDH and P5CS control the rate-limiting steps in Pro-Glu interconversion. Arabidopsis contains two isoforms of these enzymes, which may function under different conditions (Funck et al., 2010; Verslues and Sharma, 2010). In addition, plants can synthesize Pro from ornithine (Orn). This route is initiated in mitochondria, where ornithine δ-amino transferase (OAT) mediates transamination of Orn into GSA/P5C (Verslues and Sharma, 2010; Liang et al., 2013). Mitochondrial P5C is a common product of ProDH and OAT activities, and may be either transformed into Glu by P5CDH initiating the Glu pathway (Funck et al., 2008), or transferred to cytosol to generate Pro by P5CR (Miller et al., 2009). Then, coordination of P5CDH and P5CR determines the use of P5C under stress (Figure 1).

Enzymes controlling Pro metabolism are regulated at different levels (Fichman et al., 2014; Kavi Kishor and Sreenivasulu, 2014). In general, OAT, P5CS, and ProDH are subject to transcriptional control, whereas P5CR and P5CDH may be regulated at both, transcriptional and post-transcriptional levels. P5CS displays allosteric inhibition by endproduct and is affected by alternative splicing. P5CDH is post-transcriptionally regulated by *cis* antisense transcripts, and P5CR is sensitive to redox regulation (Giberti et al., 2014). Coordination of synthesis and catabolism is much more complex and has been less studied under both, physiological and stress conditions. In some cases both routes may be activated together. *P5CS* (Savoure et al., 1995), *ProDH* (Nakashima et al., 1998), and *P5CDH* (Deuschle et al., 2004) may be induced in parallel in reproductive organs. Similarly, *P5CS2, P5CR*, and *ProDH1* increase their expression in meristematic tissues such as root tip, shoot apex, and inflorescences (Kavi Kishor and Sreenivasulu, 2014). In addition, under some stress conditions genes from synthesis and catabolism are simultaneously induced (Fabro et al., 2004; Kaplan et al., 2007; Sharma and Verslues, 2010; Senthil-Kumar and Mysore, 2012). Recently, a shortcut connecting synthesis and catabolism by coupling ProDH and P5CR activities (Pro/P5C cycle) was suggested to operate under stress (Miller et al., 2009; Lv et al., 2011; Monteoliva et al., 2014). However, it is worth noting that the function of this cycle is sustained in the exclusive location of P5CDH and P5CR in mitochondria and cytosol, respectively, as well as in the existence of P5C transporters in plants.

As observed, Pro metabolism is subject to a complex control and its alteration may have different consequences. The Glu-Pro conversion consumes NADPH in cytosol and chloroplasts and may thus alleviate photoinhibition, as well as repression of the oxidative pentose phosphate pathway. The Pro-Glu pathway provides reducing equivalents for mitochondrial oxidative phosphorylation (Verslues and Sharma, 2010; Liang et al., 2013). Therefore, Pro-Glu interconversion couples oxidation of the cytosolic NADPH/NADP^+^ pool with mitochondrial activity. In turn, the Pro/P5C cycle shuttles reductants into mitochondria and promotes accumulation of reactive oxygen species (ROS) probably due to hyper-activation of ProDH (Ben Rejeb et al., 2014). Given this scenario, the activity of P5CDH seems to be crucial to define the fate of P5C (synthesis of Glu or Pro), the degree of Pro oxidation, and the level of ROS. Moreover, P5CDH can provide plasticity to turn on or off synthesis and degradation routes at different stages of stress. Despite this, the effects of this enzyme on the coordination of Pro metabolic pathways have been poorly studied.

Plants that trigger Pro consumption as a first response to stress, may replenish the amino acid pool by synthesis or transport. While Glu is considered the main source of Pro under stress, the Orn pathway provides Pro at early developmental stages and under high nitrogen conditions where it allows transfer of nitrogen from Arg to other amino acids (Verslues and Sharma, 2010; Liang et al., 2013). In addition, Orn can also be used to synthesize Pro under hostile conditions. *N. plumbaginifolia* plants over-expressing Arabidopsis OAT increase Pro levels in response to osmotic stress (Roosens et al., 2002). Curiously, different effects have been reported for this enzyme under salinity. Radish cotyledons activate OAT by treatment with NaCl (100–250 mM) in a dose-dependent manner, and reduce salt-induced Pro accumulation (six-fold) in response to OAT inhibitors (Hervieu et al., 1995). OAT activation also accompanies Pro accumulation (12-fold) in cashew leaves treated with 100–400 mM NaCl (Da Rocha et al., 2012). In contrast, the absence of OAT does not affect Pro increase (four-fold) in Arabidopsis plants treated with 25–100 mM NaCl (Funck et al., 2008). Interestingly, although OAT may synthesize Pro under some particular adverse conditions, it is unknown how this is achieved (*via* P5CDH/P5CS/P5CR or P5CR). Similarly, the traits that determine the use of Glu or Orn as Pro precursors, and the role of P5CDH on coordination of the different Pro pathways under stress deserve further characterization. The exhaustive study of Pro metabolism under these circumstances may contribute to identify metabolic and physiological features associated to stress tolerance.

In the present work, we evaluated whether Glu and Orn are used to regenerate Pro in tissues that initially consume Pro as a result of Pro supply, or *Pst-AvrRpm1* infection. Using wild-type and *p5cdh* mutant plants we assessed how P5CDH affects the catabolic and biosynthetic pathways under these conditions. To this end, we monitored the expression of Pro metabolic genes and the levels of Pro, Orn, and Glu at different stages of plant treatment. Our results indicated that both plants trigger Pro synthesis after initial Pro degradation apparently activating different metabolic circuits.

## Materials and methods

### Plant growth and pathogen infection

Arabidopsis Col-0 (wild-type) and *p5cdh* mutant (Salk_021026) seeds were obtained from ABRC (Arabidopsis Biological Research Center, Ohio, USA). Plants were grown on GM-agar medium (GM with 30 mM sucrose) for 10 days, and then transferred to soil for 6–8 weeks in growth chamber under 12/12 light-dark photoperiod at 22°C. *Pst-AvrRpm1* was grown in King's B agar medium (2% proteose peptone, 1% glycerol, 1.5 g l^−1^ K2HPO4, 1% agar) with kanamycin (50 mg/ml) and rifampicin (100 mg/ml), and used to infiltrate leaves at 1 × 10^7^ cfu/ml as described by Cecchini et al. (2011).

### Pro feeding assay

Leaves of 6–8 weeks old Col-0 and *p5cdh* plants were excised with a razor blade and transferred to eppendorf tubes containing 300 μl of 20 or 50 mM Pro solution (Sigma-Aldrich, St Louis) avoiding formation of air bubbles, and maintained under the growth conditions indicated above. To determine Pro uptake we measured the volume of feeding solution remaining in test tubes at 0, 2, 6, 10, and 24 h post-incubation (4 replicates per genotype). Tubes containing no leaves were used to evaluate and discount evaporation. Aliquots (5 μl) extracted at 0, 6, and 24 h were used to determine Pro content (Bates et al., 1973). After feeding, leaves were rinsed with distilled water and transferred to wet chamber without water uptake.

### Cell death analysis

Trypan blue staining (Pavet et al., 2005) was used to detect dead cells in leaves fed for 24 h with water (control) or Pro solutions (50, 100, or 150 mM).

### Quantification of amino acids by HPLC

HPLC was used to quantify Pro, Glu, and Orn in leaf extracts after derivatization with 6-aminoquinolyl-*N*-hydroxysuccinimidyl carbamate as reported by Monteoliva et al. (2014). Amino acids separation was carried out using a Zorbax C18 Plus column (100 × 4.6 mm, 3.5 μm) on a quaternary HPLC system (Hewlett Packard series 1100) with: (A) sodium acetate buffer 140 mM pH 5.8 and 7 mM triethylamine; (B) acetonitrile and (C) water. The gradient elution was 0.01 min 100% A; 0.50 min 99% A + 1% B; 27.5 min 91% A + 9% B; 28.50 min 89% A + 11% B; 44.50 min 82% A + 18% B; 47.5 min 60% B + 40% C; 50.5 min 100% A. The equipment was coupled to a fluorescent detector (excitation at 300 nm and emission at 400 nm). Retention times and quantification were determined using external standards. Values are expressed as nmol/g FW.

### Gene expression

cDNA was synthesized from 2 μg RNA as previously described (Cambiagno et al., 2015). Semi-quantitative PCR was developed with primers and conditions indicated in Supplementary Table 3. *GapC* was used as housekeeping gene to control equal amount of cDNA in each reaction. qPCR was performed with Hot Start DNA Polymerase (Biodynamics), SYBR green dye, and dNTPs. Primers were used at the annealing temperature indicated in Supplementary Table 3. Reactions were developed as follows: 10 min at 95°C; 40 cycles of 35 s at 95°C, 30 s of annealing, and 35 s at 72°C. Reaction efficiency (E) was in the range 81–91% for all analyzed genes, including *UBQ5* (*At3g62250*) used as internal control. Relative transcript levels (RTL) were calculated using the Pfaffl equation as follows: RTL = E_target_^−ΔΔCt (treated,basal)^/E_*UBQ*5_^−ΔΔCt (treated,basal)^.

### Protein levels and prodh activity

Western blots were performed with polyclonal antibodies as previously described (Monteoliva et al., 2014). *In vitro* ProDH activity was determined by the method reported in Monteoliva et al. (2014) with two minor modifications: (i) grinding was performed with an automated tissue disruptor; (ii) ^14^C-Pro (NEC Perkin Elmer; 266 mCi/mmol) was used as substrate. Two aliquots per sample (± T4C; L-thiazolidine-4-carboxylic acid) were used. In the activities here reported the values of T4C-treated aliquots have been subtracted.

## Results

### Pro uptake in detached leaves

We wondered if P5CDH affects Pro metabolism in tissues transiently exposed to Pro. To test this, wild-type (Col-0) and *p5cdh* tissues were treated with Pro solutions (feeding phase) and then deprived of the amino acid (recovery phase). These treatments would initially activate ProDH (Kiyosue et al., 1996; Satoh et al., 2002), and then promote Pro consumption and eventually re-activation of Pro synthesis.

The studies were conducted on detached leaves of analogous developmental stage that were excised from plants of similar age (6–8 weeks). This model, at difference of whole plants, allows studying homogeneous responses unaffected by transport of substances between organs. In the feeding phase Pro was supplied through the leaf petiole, and in the recovery phase leaves were maintained in a humid chamber without water uptake. The experiments described below differ in the concentration of Pro solutions, and the periods of feeding or recovery.

**Figure 2:**
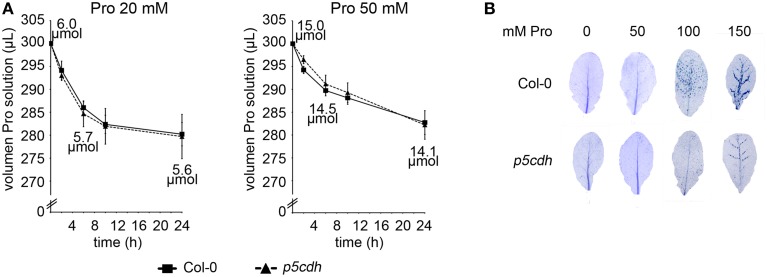
**Uptake and toxicity of Pro solutions in Col-0 and *p5cdh* detached leaves**. **(A)** Detached leaves were incubated with Pro solutions for 24 h (300 μl, 20 or 50 mM Pro). Pro uptake was evaluated by determining the volume of solution remaining in test tubes at 2, 6, 10, and 24 h after feeding. Data represent the average ± SE of 2 experiments (4 leaves each). **(B)** Trypan blue staining of detached leaves exposed for 24 h either to Pro solutions (50, 100, 150 mM) or water (0 mM).

To start characterizing the experimental system, we assessed Pro uptake in both genotypes. For this purpose, aliquots of the Pro solutions used to feed individual leaves were taken at 2, 6, 10, and 24 h post treatment (hpt) to determine Pro content. In addition, volume solution was checked at these stages. Pro 20 and 50 mM solutions conserved their original concentration at 24 hpt (20.2 ± 0.2 or 49.9 ± 1.9 mM Pro, respectively). Sustained volume reduction was detected in all assays, and was faster for incubations with 20 mM Pro (Figure 2A). Solution volumes were identical in tubes holding different genotypes, analyzed at the same time point and Pro concentration. Hence, in this assay Col-0 and *p5cdh* leaves showed no differences in Pro uptake.

The estimated amino acid content at 0, 6 and 24 hpt with each Pro solution is informed on Figure 2A. Based on these values, and the average weight of a leaf (30 mg), we estimated that individual leaves could incorporate up to 400 or 900 nmol Pro at 24 hpt when fed with 20 or 50 mM Pro, respectively.

In our hands, Col-0 and *p5cdh* leaves did not show significant damage at 24 hpt with 50 mM Pro (Figure 2B). Nevertheless, higher Pro concentrations (100 or 150 mM Pro) triggered perivascular cell death in both genotypes. Curiously, cell death was less pronounced on *p5cdh* than wild-type leaves.

### Changes in pro levels during feeding and recovery

**Figure 3:**
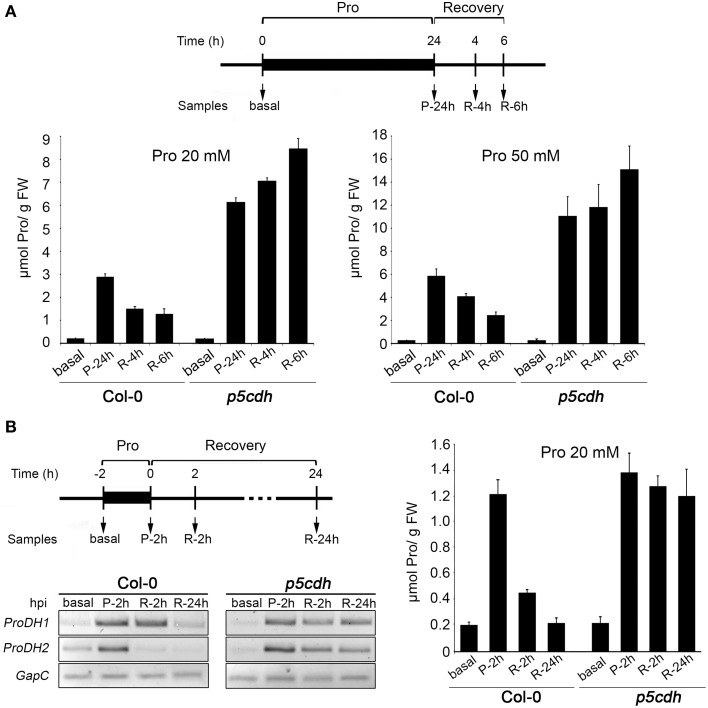
**Pro levels and *ProDH* expression in wild-type and *p5cdh* detached leaves treated with Pro**. **(A)** Leaves were fed with 20 or 50 mM Pro during 24 h, and then deprived of the amino acid for 6 h (recovery) to evaluate samples at different stages of treatment (top). Graphics compare the Pro levels in both genotypes (bottom). **(B)** Leaves fed with 20 mM Pro for 2 h, and deprived of the amino acid for 24 h, were used to determine Pro levels and *ProDH* expression at different stages (top). Gene expression was studied by sq-RT-PCR using *GapC* as internal control (bottom). Pro was quantified by colorimetric assays (right). In each case, one representative experiment from three independent assays is shown. Data represents the average ± SE of 3 leaves per time point. Pro content displayed significant differences (*p* < 0.001 by *t*-test) between treated samples and basal condition in both genotypes, except for the R-24h, Col-0 sample that was similar to control **(B)**.

Next, we used a colorimetric assay (Bates et al., 1973) to quantify the free Pro content in leaves. Leaves exposed to Pro (20 or 50 mM, 24 h) and transferred to humid chamber (6 h), were analyzed at basal condition (basal), 24 h post-feeding (P-24h), and 4 and 6 h of recovery (R-4h, R-6h) (Figure 3A).

At the stage P-24h, Col-0 leaves increased 14 or 27 times the basal Pro levels, reaching 2.9 ± 0.1 or 5.8 ± 0.6 μmol Pro/g FW in response to 20 or 50 mM Pro, respectively (Figure 3A). At the same stage, *p5cdh* leaves increased 27 or 41 times the amino acid content (6.0 ± 0.2 or 11.0 ± 1.7 μmol Pro/g FW for 20 or 50 mM Pro, respectively), almost doubling the levels of Col-0 leaves.

Then, Pro was higher in leaves exposed to higher Pro concentrations, and for a given solution was greater in *p5cdh* than Col-0 leaves. The fact that Pro uptake was similar in both leaves (Figure 2A), but Pro leaf content was higher in *p5cdh* (Figure 3A) suggested that during feeding Pro may be consumed more efficiently in Col-0. Supporting this possibility, differences in tissue Pro were manifested during recovery, when Col-0 sharply reduced the Pro content (half levels at R-6h *vs* P-24h), while *p5cdh* did not consume it, and even manifested a slight trend to increase the amino acid level (Figure 3A).

To test if ProDH was activated at the feeding phase, leaves were incubated with 20 mM Pro for only 2 h, and then transferred to humid chamber (Figure 3B). Samples obtained at basal condition, 2 h post-feeding (P-2h), and 2 and 24 h of recovery (R-2h, R-24h), were used to determine Pro content and *ProDH* genes expression. At P-2h, Pro increased at the same extent in Col-0 and *p5cdh* leaves (6 fold compared to basal condition), reaching 1.2 ± 0.1 or 1.4 ± 0.1 μmol/g FW, respectively (Figure 3B), and *ProDH1* and *ProDH2* genes displayed similar activation. During recovery, Col-0 leaves initially lost *ProDH2* induction (R-2h), and then *ProDH1* activation (R-24h). In parallel, these leaves reduced Pro to almost recover its basal content at R-24h (0.219 ± 0.041 Pro/g FW). In contrast, *p5cdh* leaves maintained *ProDH1* and *ProDH2* activation until R-24h, showing no significant reduction in the Pro content (14% at R-24h vs. P-2h) (Figure 3B).

Consequently, 2 h treatment with Pro 20 mM was sufficient to induce *ProDH1* and *ProDH2* in Col-0 and *p5cdh* leaves, but insufficient to generate differential Pro content in these genotypes. Since this kind of differences were manifested at 24 h post feeding (P-24h, Pro 20 or 50 mM; Figure 3A), it is likely that they result from processes not yet triggered at 2 h post feeding.

### Metabolic alterations triggered by exogenous pro

**Figure 4:**
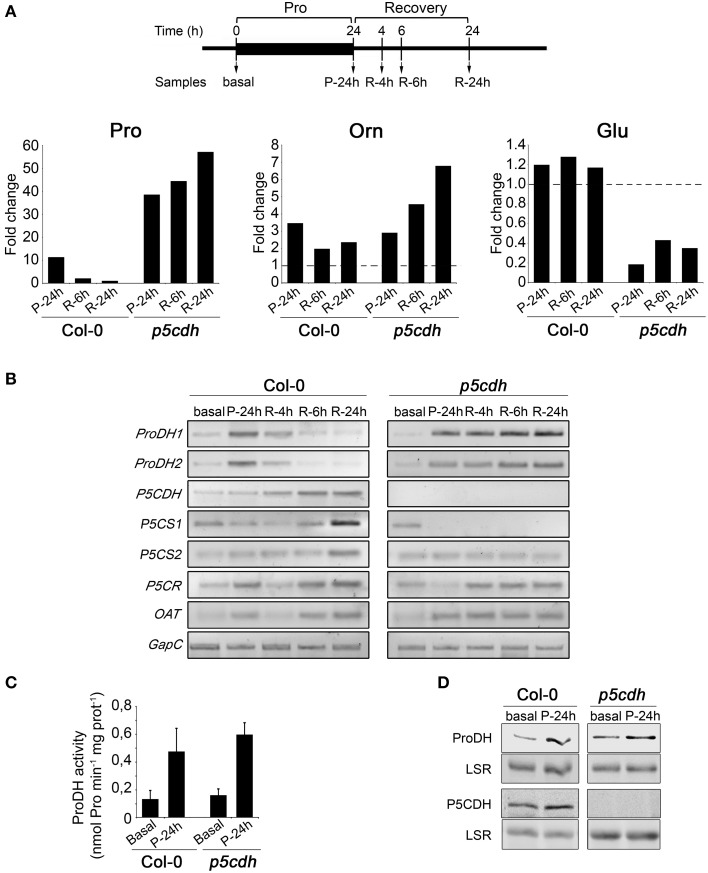
**Metabolic changes induced by exogenous Pro in detached leaves of wild-type and *p5cdh* plants**. **(A)** Leaves were incubated with 20 mM Pro (24 h) and then deprived from the amino acid (24 h) to take samples at different treatment stages (top). Pro, Glu, and Orn levels were determined by HPLC (Supplementary Table 1) and the ratio between their content in treated and basal conditions was depicted (bottom). **(B)** Expression of genes of Pro metabolism evaluated as described in Figure 3B. **(C)** ProDH activity in total extracts of leaf samples taken at basal and P-24h stages. Values are expressed as nmol of Pro min^−1^ mg protein^−1^. Each value is mean ± SE of two biological replicates. **(D)** ProDH and P5CDH content at basal and P-24h conditions examined by Western blot with polyclonal antibodies. LSR, large subunit of rubisco.

*p5cdh* leaves seem to accumulate Pro after being deprived of the amino acid (R-6h vs. P-24h; Figure 3A). To test if this response was strengthened along recovery, we preserved the feeding period (24 h) and extended the recovery phase (24 h) using incubations with 20 mM Pro. *p5cdh* and Col-0 leaves were analyzed in parallel at basal, P-24h, R-4h, R-6h, and R-24h stages (Figure 4A). To comprehensively assess the Pro metabolic routes altered under this condition, we evaluated amino acids content and expression of genes of Pro metabolism (*ProDH, P5CDH, P5CS, P5CR*, and *OAT*).

We conducted HPLC studies to unequivocally determine the leaf Pro content. By adapting this assay we also quantified Glu and Orn in the same samples. Pro, Orn and Glu values are reported in Supplementary Table 1, by indicating significant differences along treatment for each compound in the same genotype. All amino acids altered their levels on at least one condition. To better visualize these changes, we expressed the fold change of a compound in the test sample (P-24h, R-6h, R-24h) relative to control sample (basal) (Figure 4A).

Changes in Pro levels detected by HPLC assays were consistent with those previously observed by colorimetric reactions. That is, at P-24h *p5cdh* contained higher Pro levels than Col-0 samples and during recovery, *p5cdh* accumulated Pro while Col-0 reduced it. The Pro increase occurred in *p5cdh* during recovery was intensified along this phase (Figure 4A).

On the other hand, Glu was not significantly altered in Col-0 leaves, but was clearly reduced in *p5cdh* at all phases analyzed (reduction of 81% at P-24h, and 65% at R-24h, compared to basal levels). As Glu is a central intermediate for synthesis of amino acids, this reduction may derive from its conversion into other derivatives different from Pro, such as Arg, GABA, Gln, or inhibition of the routes that replenish this amino acid (Forde and Lea, 2007).

Curiously, Orn increased in Col-0 and *p5cdh* leaves after feeding (3.5 and 2.9 times, respectively, compared to basal condition). Latter, during recovery, Orn was reduced to half values in Col-0, while increased in *p5cdh* (2.3 fold at R-24h, compared to P-24h) (Supplementary Table 1, Figure 4A). In summary, the genotypes kept their trends to either consume (Col-0) or accumulate (*p5cdh*) Pro, and these variations were accompanied by similar changes in Orn (reduction in Col-0, increase in *p5cdh*), but did not correlate with changes in Glu levels.

As mentioned, we conducted gene expression analysis with the same set of samples (Figure 4B). Col-0 leaves accumulated *ProDH1* and *ProDH2* transcripts at P-24h, and reverted gene induction at R-6h, when Pro doubled its initial content (Supplementary Table 1). Then, these leaves activated *P5CDH* (R-4h), and *P5CS1* (R-6h), displaying maximal *P5CS1* expression at R-24h when Pro recovered its basal level (Figure 4B, Supplementary Table 1). Moreover, at P-24h, R-6h, and R-24h, *P5CR* and *OAT* were induced in these samples. These results suggest that once Col-0 leaves stimulate Pro synthesis after consuming the amino acid incorporated from the medium involving P5CS and OAT, and P5CR activities. At this condition, the synthesis of Pro from Glu and Orn, converging on P5C, could help maintain the Pro basal level. Our experimental scheme did not inform whether this route would generate accumulation of Pro at longer times.

The *p5cdh* leaves exhibited a different behavior. *ProDH1* and *ProDH2* were also induced at P-24h, but reinforced their activation along recovery, reaching maximal expression at R-24h (Figure 4B). Thus, *ProDH* activation accompanied Pro increase in *p5cdh* tissues (Figure 4; Supplementary Table 1). From feeding onwards, *P5CS1* became repressed and *P5CS2* maintained basal expression. Besides this, *OAT* and *P5CR* was induced at P-24h or R-4h, respectively. Thus, the Pro increase detected in *p5cdh* leaves would not derive from the P5CS pathway. Alternatively, this increase might result from consecutive transformation of Orn into P5C and Pro, by OAT and P5CR.

RT-qPCR assays confirmed the expression pattern of *P5CS1, P5CS2, P5CR*, and *OAT* genes at basal, P-24h and R-24h stages in Col-0 and *p5cdh* leaves (Supplementary Figure 1). The quantification of ProDH activity (Monteoliva et al., 2014) in Col-0 and *p5cdh* samples at basal and P-24h stages, indicated that Pro treatment activates the enzyme to similar extent in both plants (4.1 and 3.7 fold for Col-0 and *p5cdh*, respectively) (Figure 4C). This was consistent with the increase in the ProDH content detected by Western blot in Col-0 and *p5cdh* samples at P-24h (Figure 4D). In addition, we observed that Col-0 leaves also increased P5CDH at this stage.

### Metabolic alterations triggered by pathogen infection

Arabidopsis plants infected with *Pst-AvrRpm1* initially activate ProDH and later induce *P5CS* expression accumulating Pro, suggesting that early Pro consumption stimulates the Glu biosynthetic pathway (Fabro et al., 2004; Cecchini et al., 2011; Monteoliva et al., 2014). To investigate how P5CDH affects the second process, we analyzed the behavior of wild-type and *p5cdh* plants 72 h post infection (hpi) with *Pst-AvrRpm1*. In addition, we assessed if OAT could mediate Pro synthesis under this condition. These issues, as well as the overall expression of Pro metabolism genes have not been evaluated under this condition.

**Figure 5:**
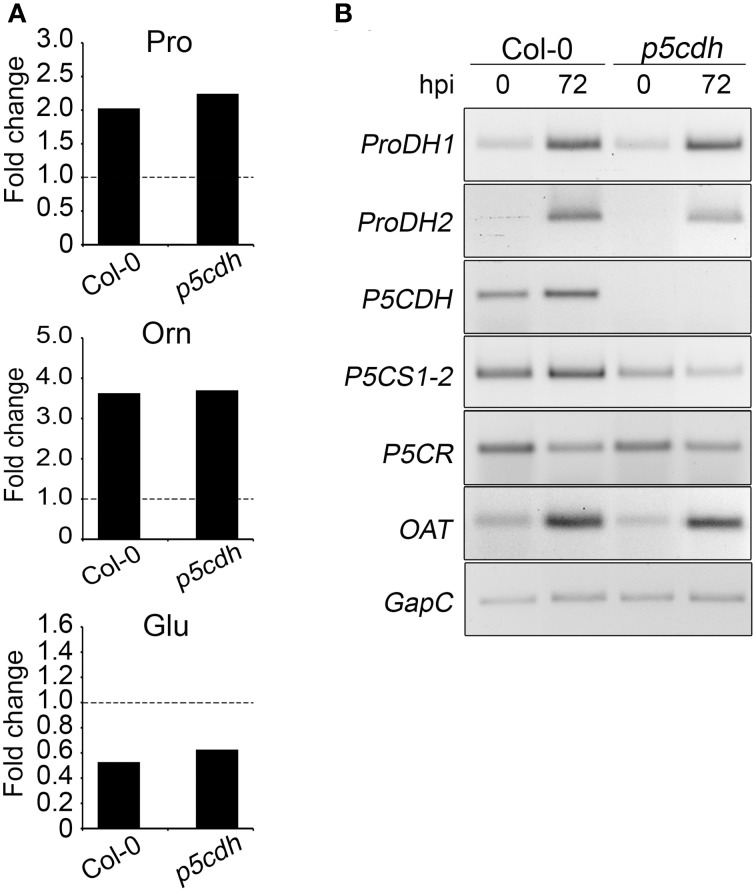
**Metabolic changes induced by *Pst-AvrRpm1* infection in wild-type and *p5cdh* plants**. **(A)** HPLC was used to determine the Pro, Glu, or Orn content in uninfected and infected tissues (72 h post-infection, hpi) (Supplementary Table 2). Fold changes of amino acid in infected and basal conditions are here informed. **(B)** Expression of genes of Pro metabolism evaluated at basal condition and 72 hpi as described in Figure 3B.

Healthy and infected leaves were used to quantify Pro, Orn, and Glu by HPLC, and assess the expression of Pro metabolism genes. Pathogen treatment similarly affected the amino acids level in both plants, where Pro content was doubled, Orn increased 3.6 times and Glu was reduced (47% in Col-0 and 37% in *p5cdh*) (Supplementary Table 2; Figure 5A). This indicated that P5CDH was not required to adjust Pro, Orn, and Glu levels at late stages of HR.

We confirmed that Col-0 plants induced *ProDH* and *P5CS* genes at 72 hpi, as previously reported (Fabro et al., 2004), increasing as well the expression of *OAT* (Figure 5B). Conversely, although infected *p5cdh* plants activated *ProDH* and *OAT* expression, they slightly repressed the expression of *P5CS*. Consequently, in advanced stages of HR wild plants could use Glu as Pro precursor while the mutant, lacking *P5CS* induction, would not stimulate this particular pathway. The activation of *OAT* occurring in both plants suggests that P5CDH is not required to generate P5C from Orn during the interaction of Arabidopsis with *Pst-AvrRpm1*.

## Discussion

### Responses of detached leaves to exogenous pro

Most studies evaluating the effect of exogenous Pro in Arabidopsis, supply the amino acid by the root (Kiyosue et al., 1996; Nakashima et al., 1998; Nanjo et al., 1999, 2003; Hellmann et al., 2000; Mani et al., 2002; Satoh et al., 2002; Deuschle et al., 2004; Miller et al., 2009; Funck et al., 2010; Sharma and Verslues, 2010). Root and shoot can differentially perceive, transport, or metabolize the amino acid, and moreover, leaves from distinct developmental stages can regulate Pro metabolism in alternative manners (Lehmann et al., 2010; Kavi Kishor and Sreenivasulu, 2014). Unlike this, our experimental model uses leaves of similar age and developmental stage that synchronously assimilate Pro and can trigger more homogeneous responses. Then, detached leaves could be useful for detection of slight changes that might go unnoticed in the whole plant system. Under the conditions tested here, Col-0 and *p5cdh* leaves assimilated Pro in similar way without saturating their absorption capacity, as the amino acid content increased with longer incubation periods or more concentrated solutions. Therefore, the responses here described likely correspond to tissues that actively incorporate and metabolize exogenous Pro. In support of this, Pro consumption was already detected at the feeding phase in Col-0 leaves.

Detached leaves fed for 24 h with 50 mM Pro accumulated similar amino acid levels (5.8 and 11.0 μmol/g FW for Col-0 and *p5cdh*, respectively) than whole plants exposed 48 h to 100 mM Pro (10 and 35 μmol/g FW for Col-0 and *p5cdh* respectively; Miller et al., 2009). These leaves did not manifest significant lesions under the above conditions, although they induced cell death in perivascular tissues in response to higher Pro concentrations (100–150 mM). Pro also induced leaf cell death in plants sprayed for 3 consecutive days with 20 mM solution (Deuschle et al., 2004), indicating that in photosynthetic tissues its toxicity is independent of the route of entry (phloem or epidermis). Curiously, Pro treatment produced less damage in *p5cdh* than Col-0 leaves (Figure 2B). As the mutant accumulated higher Pro content, then Pro levels and cell death symptoms would not be directly correlated. The finding of more cell death in the mutant contrasts with the effect reported for the amino acid supplied by root to seedlings of these genotypes (Deuschle et al., 2004; Funck et al., 2010). Therefore, we cannot discard that *p5cdh* plants exhibit dissimilar sensitivity to exogenous Pro in roots and leaves.

### Pro metabolism changes induced by exogenous pro

**Figure 6:**
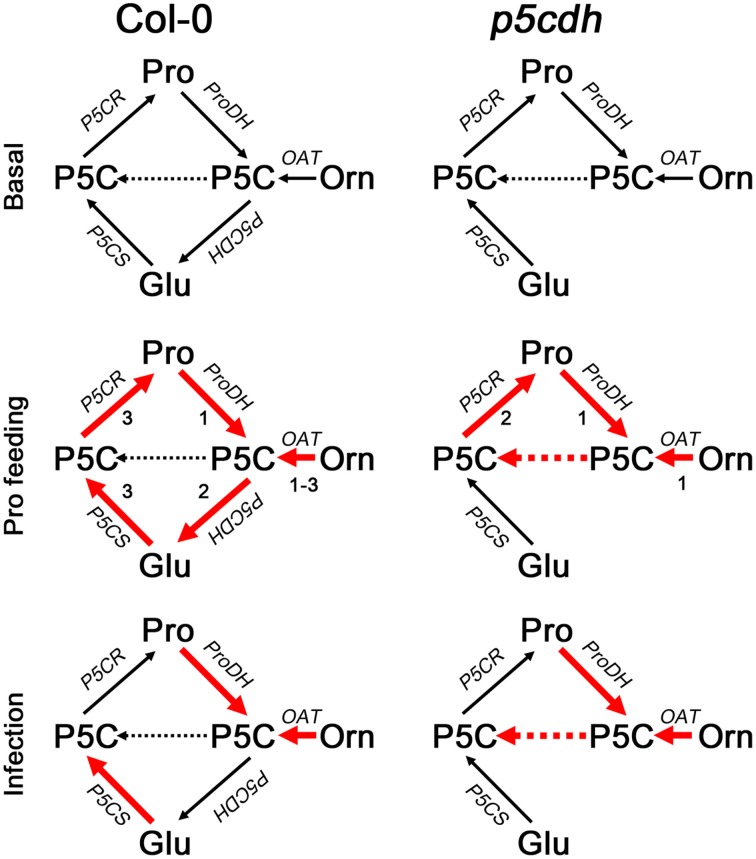
**Model of Pro metabolism in wild-type and *p5cdh* mutant plants**. Metabolic routes are represented at basal condition (top), and after treatment with exogenous Pro (middle) or *Pst-AvrRpm1* (bottom). Red lines represent gene activation. Numbers indicate the timing of gene induction according to results from Figure 4B. For further information see Discussion section.

Col-0 and *p5cdh* leaves treated with exogenous Pro displayed similar ProDH activation (Figure 4C) accompanied by gene induction (Figure 4B) and protein accumulation (Figure 4D). This increase in ProDH activity (3–4 fold) was similar to that reported for Arabidopsis cells treated with 50 mM Pro during 21 h (4–5 fold; Schertl et al., 2014). However, this treatment triggers different metabolic changes in both plants (Figures 4A,B, 6). Wild-type leaves consumed the excess of Pro, to then increase the *P5CS1* expression and apparently stimulate the synthesis of Pro from Glu. In contrast, *p5cdh* leaves maintained *ProDH* and *P5CR* induction, apparently activating the Pro/P5C cycle. Curiously, these leaves accumulated Pro after being removed from the feeding solution. This response did not seem to be caused by massive protein degradation as it was not accompanied by tissue damage or general rise in free amino acids. Such Pro increase was manifested after prolonged feeding (P-24h but not P-2h), and was accentuated throughout the recovery phase (R-24h vs. R-6h). The activation of *OAT*, but not *P5CS*, detected under this condition suggested that Pro may derive from Orn (Figure 4B). As the mutant cannot use P5C in the catabolic pathway, it is possible that OAT provides P5C into the biosynthetic route leading to Pro accumulation (Figure 6). The *p5cdh* tissues that induced *OAT* (Figure 4A) accumulate Pro (Supplementary Table 1). Moreover, *in vitro* assays suggested that OAT activity was not inhibited by high Pro levels (Sekhar et al., 2007). Note that the Orn concentration found at P-24h in *p5cdh* leaves was 100 times lower than that of Pro, and thereafter Orn and Pro increased together (Supplementary Table 1). This suggests that Orn accumulation corresponds to the amino acid pool that feeds Pro synthesis. In this case, Pro and Orn may not increase in similar manner, since their synthesis and degradation could be altered differently (i.e., greater increase in Orn to Pro conversion, than in Pro degradation). Parallel increases of Orn and Pro have been also observed in cashew plants treated with NaCl, which activated OAT and curiously repressed P5CDH (Da Rocha et al., 2012). It is important to note that our results are insufficient to establish that *p5cdh* leaves synthesize Pro from Orn during recovery. Additional studies will be required to formally demonstrate this assumption. In addition, it is not clear whether Col-0 leaves synthesize Pro from Orn at the recovery phase, since they reduce the Orn pool and slightly increase the *OAT* transcripts. We currently ignore which amino acid may act as Orn precursor during the recovery phase. Since Col-0 and *p5cdh* leaves activate the expression of the *ARG1* (arginase) gene at R-24h (Supplementary Figure 1), it is possible that Arg is used to this end.

A curious result was the repression of *P5CS1* caused by Pro treatment in *p5cdh* leaves (Figure 4B). This response may not be caused by Pro accumulation (6 μmol/g FW Pro; Figure 3A), since Pro-treated Col-0 leaves reaching similar amino acid content did not reduce *P5CS1* expression (not shown). Furthermore, *p5cdh* seedlings repressed *P5CS* during recovery from drought (Miller et al., 2009), suggesting that the absence of P5CDH would somehow alter transcriptional regulation of *P5CS1* under stress.

### Pro metabolism changes induced by infection with *Pst-AvrRpm1*

Arabidopsis plants infected with *Pst-AvrRpm1* initially activate ProDH, to then induce *P5CS* and accumulate Pro (Fabro et al., 2004; Cecchini et al., 2011; Monteoliva et al., 2014). To learn more about the Pro metabolic routes that become altered at late stages of infection, we compared the behavior of *p5cdh* and Col-0 plants. At 72 hpi both genotypes modified the Pro, Orn, and Glu levels in similar way, but they used slightly different alternatives to generate these changes.

Both plants activated *OAT* and increased Orn and Pro. As described before, this may suggest that Orn is used as a Pro precursor. The rise in Orn taking place in infected tissues may derive from activation of arginase, which generates Orn from Arg, as the enzyme is enriched in the mitochondrial fraction of Arabidopsis tissues infected with *Pst-AvrRpm1* (Jones et al., 2006). Interestingly, *N. benthamiana* tissues infected with *Pseudomonas syringae* pv. *tomato* T1 may also synthesize Pro from Orn, since they require *OAT* for normal development of HR (Senthil-Kumar and Mysore, 2012). Here again, the notion that OAT feeds Pro synthesis in *p5cdh* infected leaves is consistent with activation of the Pro/P5C cycle in these tissues (Monteoliva et al., 2014).

Reduction of Glu accompanies Pro increase in both plants, but is only associated with induction of *P5CS* gene in wild plant, suggesting that this reduction may not necessarily reflect the activation of this pathway.

Therefore, Col-0 and *p5cdh* plants treated with *Pst-AvrRpm1* would combine differently the anabolic and catabolic routes to generate similar Pro increases. One possibility is that both biosynthetic routes (P5CS and OAT) and the complete catabolic route (ProDH, P5CDH) are combined in Col-0, while one anabolic route (OAT) and partial Pro oxidation work in *p5cdh* (Figure 6).

In summary, both experimental systems enable ProDH induction and apparently subsequent Pro synthesis, triggering less pronounced amino acid changes at late infection stages than in response to Pro treatment. *p5cdh* tissues seem to maintain ProDH participating in the Pro/P5C cycle, while feeding Pro synthesis with the P5C derived from OAT activity. In contrast, wild-type tissues seem to use OAT and/or P5CS for Pro synthesis and ProDH and P5CDH for Pro catabolism. These results demonstrate that under stress P5CDH may indeed affect the fate of P5C, and the routes of Pro synthesis (OAT or P5CS), while the absence of this enzyme could reduce the ability of plants to induce *P5CS* expression.

### Conflict of interest statement

The authors declare that the research was conducted in the absence of any commercial or financial relationships that could be construed as a potential conflict of interest.

## Supplementary material

Supplementary Table 1 **Amino acid content in detached leaves from wild-type and *p5cdh* plants treated with exogenous Pro**.

Supplementary Table 2 **Amino acid content in wild-type and *p5cdh* plants infected with *Pst-AvrRpm1***.

Supplementary Table 3 **Primers and conditions used for RT-sqPCR and RT-qPCR**.

Supplementary Figure 1 **Expression level of Pro metabolism genes in wild-type and p5cdh leaves treated with 20 mM Pro determined by RT-qPCR**. The samples used in this study are those shown in Figure 4B. Relative transcript levels of different Pro metabolism genes **(A–E)** were calculated as described in Materials and Methods. Letters indicate statistically significant differences respect to the basal sample (a: *p* < 0.05; b: *p* < 0.01, by *t*-test).

## Abbreviations

Glu: glutamic acid
GSA: glutamate-semialdehyde
HPLC: high performance liquid chromatography
HR: hypersensitive response
OAT: ornithine δ-aminotransferase
Orn: ornithine
P5C: Δ1 pyrroline-5-carboxylate
P5CDH: Δ1 pyrroline-5-carboxylate dehydrogenase
P5CR: Δ1 pyrroline-5-carboxylate reductase
P5CS: Δ1 pyrroline-5-carboxylate synthase
Pro: proline
ProDH: proline dehydrogenase
*Pst-AvrRpm1*: *Pseudomonas syringae* pv. *tomato* DC3000 *AvrRpm1*
ROS: reactive oxygen species.
