# Supplementary material for: P5CDH affects the pathways contributing to Pro synthesis after ProDH activation by biotic and abiotic stress conditions
Source: Front Plant Sci. 2015 Jul 28;6:572. doi: 10.3389/fpls.2015.00572 (PMC4517450; doi:10.3389/fpls.2015.00572)
Supplement: Supplementary Table 1 — Amino acid content in detached leaves from wild-type and p5cdh plants treated with exogenous Pro. [file Table1.DOCX]

**Supplementary Table 1**. **Amino acids content in detached leaves from wild type and *p5cdh* plants treated with exogenous Pro**.

Excised leaves from adult plants (6-8 weeks) were fed with 20 mM Pro during 24 h (P-24h) and then transferred to wet chamber for 6 or 24 h (R-6h, R-24h). Amino acids were quantified by HPLC. a, b and c indicate significant differences between treatments for each plant (p<0,05 by ANOVA and Tukey test). Values are expressed as nmol/g FW. Each value is mean ± SE of 3-6 independent experiments. On each experiment 3 leaves were used per time point.

|  | **Col-0** | | | |  | ***p5cdh*** | | | |
| --- | --- | --- | --- | --- | --- | --- | --- | --- | --- |
|  | Basal | P-24h | R-6h | R-24h |  | Basal | P-24h | R-6h | R-24h |
| **Pro** | 125 ± 23^a^ | 1400 ± 381^b^ | 247 ± 45^a^ | 115 ± 16^a^ |  | 213 ± 44^a^ | 8172 ± 823^b^ | 9425 ± 1088^bc^ | 12113 ± 744^c^ |
| **Orn** | 13 ± 2^a^ | 45 ± 8^b^ | 26 ± 4^ab^ | 31 ± 7^ab^ |  | 26 ± 5^a^ | 76 ± 21^ab^ | 119 ± 29^ab^ | 177 ± 26^b^ |
| **Glu** | 929 ± 177^a^ | 1113 ± 45^a^ | 1188 ± 36^a^ | 1087 ± 130^a^ |  | 1096 ± 55^a^ | 203 ± 26^b^ | 473 ± 61^c^ | 385 ± 83^bc^ |
