# Supplementary material for: P5CDH affects the pathways contributing to Pro synthesis after ProDH activation by biotic and abiotic stress conditions
Source: Front Plant Sci. 2015 Jul 28;6:572. doi: 10.3389/fpls.2015.00572 (PMC4517450; doi:10.3389/fpls.2015.00572)
Supplement: Supplementary Table 2 — Amino acid content in wild-type and p5cdh plants infected with Pst-AvrRpm1. [file Table2.DOCX]

**Supplementary Table 2. Amino acids content in wild type and *p5cdh* plants infected with *Pst*-*AvrRpm1***.

Leaves infiltrated with 1x10^7^cfu/ml *Pst-AvrRpm1*, were used to determine the amino acids content by HPLC at 72 hours post infection (hpi). Untreated leaves were evaluated in parallel as control (0 hpi). a and b indicate significant differences between treatments for each plant (p<0,05 by t-test). Values are expressed as nmol/g FW. Each value is mean ± SE of 5-6 independent experiments. On each experiment 3 leaves were used per time point.

|  | **Col-0** | |  | ***p5cdh*** | |
| --- | --- | --- | --- | --- | --- |
|  | 0 hpi | 72 hpi |  | 0 hpi | 72 hpi |
| **Pro** | 151 ± 19^a^ | 305 ± 32^b^ |  | 204 ± 20^a^ | 457 ± 89^b^ |
| **Orn** | 18 ± 3^a^ | 64 ± 12^b^ |  | 19 ± 3^a^ | 70 ± 20^b^ |
| **Glu** | 978 ± 21^a^ | 516 ± 70^b^ |  | 1087 ± 147^a^ | 681 ± 27^b^ |
