# Supplementary material for: P5CDH affects the pathways contributing to Pro synthesis after ProDH activation by biotic and abiotic stress conditions
Source: Front Plant Sci. 2015 Jul 28;6:572. doi: 10.3389/fpls.2015.00572 (PMC4517450; doi:10.3389/fpls.2015.00572)
Supplement: Supplementary Table 3 — Primers and conditions used for RT-sqPCR and RT-qPCR. [file Table3.DOCX]

**Supplementary Table 3**. **Primers and conditions used for RT-sqPCR and RT-qPCR.**

# Annealing temperature used for each pair of primers.

* For each pair of primers the optimal cycle number was selected from a linear amplification range using the following conditions: 2 μg RNA, 1 μg random hexamer primers, 200 U M-MLV reverse transcriptase for cDNA synthesis; 1 µl cDNA, 10 µM primer, 20 µl final volume for PCR. The housekeeping gene *GapC* was used as internal control.

| **Gene**  **(AGI Nº)** | **Primers** | **Ta#**  **(ºC)** | **Cycles*** |
| --- | --- | --- | --- |
| *ProDH1*  *(At3g30775)* | Fw: 5´-TGATGGAGAAAGCATCAAACGG-3´ | 60 | 31 |
|  | Rv: 5´-TCTCCTCTTAAGTTCCATCCTC-3´ |  |  |
| *ProDH2*  *(At5g38710)* | Fw: 5´-CGTCGAAGCTGCTAAAACCCT-3´ | 60 | 29 |
|  | Rv: 5´-CGTTCGATTCTTGACATCTAAG-3´ |  |  |
| *P5CDH*  *(At5g62530)* | Fw: 5´-GCGGTTCCTGGCACGGTCTT-3´ | 60 | 26 |
|  | Rv: 5´-AGTCCACATCTTCAGCGGGTAAAC-3´ |  |  |
| *P5CS1*  *(At2g39800)* | Fw: 5´-GTCGGGGTCGAAGGATTACT- 3´ | 60 | 26 |
|  | Rv: 5´-CATCGTACTTATGAGCAAGAC -3´ |  |  |
| *P5CS2*  *(At3g55610)* | Fw: 5´-CCAATATTCTCCACGTCCGCTTCTTC-3´ | 60 | 26 |
|  | Rv: 5´-GATCTCCGTCATAGTTTGTGTCTATC-3´ |  |  |
| *P5CS1-2* | Fw 5`- CGATCAAATGCTATCTTACACAAGG-3` | 60 | 25 |
|  | Rev 5`- ACAAGAAGGGTTTCCATCGCAT- 3` |  |  |
| *P5CR*  *(At5g14800)* | Fw: 5´-CACAGACCGTTCTTGGAGCTG-3´ | 62 | 26 |
|  | Rv: 5´-GTGTTGCCCGGAAAGAGCCTTT-3´ |  |  |
| *OAT*  *(At5g46180)* | Fw: 5`-TAACCGAGTGTGATCGCTGTGGAA-3` | 52 | 28 |
|  | Rv: 5`-ACCGGTGCTGTGTCCTATGGAAAT-3` |  |  |
| *GapC*  *(At3g04120)* | Fw: 5´-CACTTGAAGGGTGGTGCCAAG-3´ | 60 | 24 |
|  | Rv: 5´- CCTGTTGTCGCCAACGAAGTC -3´ |  |  |
| *UBQ5*  *(At3g62250)* | Fw: 5´-GTGGTGCTAAGAAGAGGAAGA-3´ | 60 |  |
|  | Rv: 5´-TCAAGCTTCAACTCCTTCTTT-3´ |  |  |
| *ARG1*  *(At4g08900)* | Fw: 5´-TTGCGTCCGTTGGTCTTA -3´ | 60 |  |
|  | Rv: 5´-TTGTTCCCGTCCTTCCTG -3´ |  |  |
