# Supplementary figures and images for: P5CDH affects the pathways contributing to Pro synthesis after ProDH activation by biotic and abiotic stress conditions
Source: Front Plant Sci. 2015 Jul 28;6:572. doi: 10.3389/fpls.2015.00572 (PMC4517450; doi:10.3389/fpls.2015.00572)

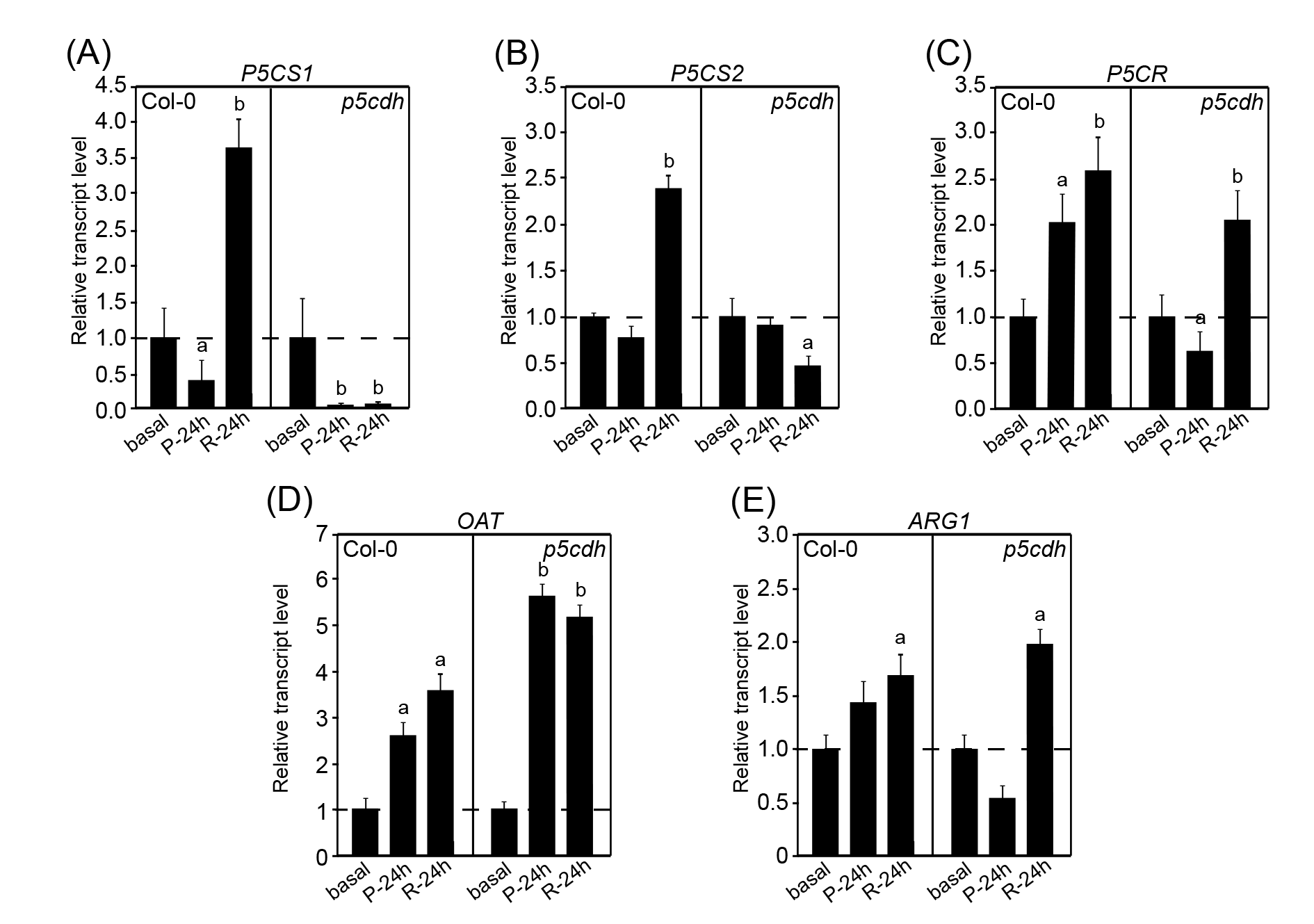

Supplement: Supplementary Figure 1 — Expression level of Pro metabolism genes in wild-type and p5cdh leaves treated with 20 mM Pro determined by RT-qPCR. The samples used in this study are those shown in Figure 4B. Relative transcript levels of different Pro metabolism genes (A–E) were calculated as described in Materials and Methods. Letters indicate statistically significant differences respect to the basal sample (a: p < 0.05; b: p < 0.01, by t-test). [file Image1.TIF]
